# All-Cause and Cause-Specific Mortality after Long-Term Sickness Absence for Psychiatric Disorders: A Prospective Cohort Study

**DOI:** 10.1371/journal.pone.0067887

**Published:** 2013-06-26

**Authors:** Anna Bryngelson, Marie Åsberg, Åke Nygren, Irene Jensen, Ellenor Mittendorfer-Rutz

**Affiliations:** 1 Department of Clinical Sciences, Karolinska Institutet, Stockholm, Sweden; 2 The Institute of Environmental Medicine, Karolinska Institutet, Stockholm, Sweden; 3 Department of Clinical Neuroscience, Karolinska Institutet, Stockholm, Sweden; Tehran University of Medical Sciences, Iran (Islamic Republic Of)

## Abstract

**Objective:**

The aim was to examine if long-term psychiatric sickness absence was associated with all-cause and diagnosis-specific (cardiovascular disease (CVD), cancer and suicide) mortality for the period 1990–2007. An additional aim was to examine these associations for psychiatric sickness absence in 1990 and 2000, with follow-up on mortality during 1991–1997 and 2001–2007, separately.

**Methods:**

Employees within municipalities and county councils, 244,990 individuals in 1990 and 764,137 individuals in 2000, were followed up to 2007 through register linkages. Analyses were conducted with flexible parametric survival models comparing sickness absentees due to psychiatric diagnoses (>90 days) with those not receiving sick leave benefit.

**Results:**

Long-term sickness absence for psychiatric disorders was associated with an increased risk of mortality due to all causes; CVD; cancer (smoking and non-smoking related); and suicide during the period 1990–2007. After full adjustment for socio-demographic covariates and previous inpatient care due to somatic and psychiatric diagnoses, these associations remained significant for all-cause mortality (Hazard ratios (HR) and 95% confidence interval (CI)): HR 1.56, 95% CI 1.3–1.8; CVD: HR 1.35, 95% CI 1.0–1.9, and suicide: HR 3.84, 95% CI 2.4–6.1. For both cohorts 1990 and 2000 estimates point in the same direction. For the time-period 2000–2007, we found increased risks of mortality in the fully adjusted model due to all causes: HR 1.47, 95% CI 1.2–1.7; CVD: HR 1.83, 95% CI 1.2–2.7; overall cancer: HR 1.33, 95% CI 1.0–1.7; and suicide: HR 2.15, 95% CI 1.3–3.7.

**Conclusion:**

Long-term sickness absence for psychiatric disorders predicted premature mortality from all-causes, cardiovascular disease, cancer, and suicide.

## Introduction

From the late 1990s there was a large increase in the proportion of individuals on long-term sickness absence who were diagnosed with a psychiatric, rather than a physical disorder, in Sweden and in other western European countries. [1]–[2] In 2009, the most common diagnoses of long-term sickness absence in Sweden were psychiatric. [1] The predominant causes for psychiatric sickness absence are depression, anxiety disorders, and stress-related disorders, while other disorders such as psychoses are much less common. [1], [3]–[4]

Psychiatric sickness absence has been shown to predict premature mortality. [5]–[9] To our knowledge, only three studies [5]–[7] have examined the relationship with cause-specific mortality. From the Whitehall study of London-based civil servants, Head and colleagues [5] found an increased risk of all-cause and cancer mortality among employees with sickness absence for psychiatric disorders. Melchior et al [6] analysed the GAZEL study of employees of the French national gas and electricity company, and found that psychiatric sickness absence was associated with an elevated risk of mortality due to cardiovascular diseases (CVD), smoking-related cancer and suicide. Mittendorfer-Rutz and colleagues [7] used a Swedish nationwide population-based material to analyse mortality outcomes of psychiatric sickness absence. The authors found an increased risk of mortality due to all-causes, suicide, cancer (both smoking and non-smoking related), as well as CVD (the latter only in men).

The above mentioned studies [5]–[7] on the relationship between psychiatric sickness absence and cause-specific mortality have used medically certified sickness absence (exceeding 7 or 14 days) as the exposure, but have not analysed long-term sickness absence of more than 3 months. Since longer sickness absence may be more closely associated with ill health than shorter sick leave [10], it seems relevant to examine if results differ when analysing long-term (>90 days) sickness absence compared with previous research on more than 7–14 days of sickness absence. Moreover, according to the Swedish Social Insurance Agency (SSIA) the number of individuals on long-term (>60 days) sickness absence increased over the period from 1990 until the first years of the 21th century in Sweden. [4] Additionally, the proportion of long-term (>60 days) sickness absentees for psychiatric disorders among all long-term sick-listed increased over the last two decades. [1], [11] Therefore it seems relevant to examine if a potential association between psychiatric long-term sickness absence and mortality holds for two different cohorts in 1990 and 2000. Since deaths are rare events, a long follow-up period of 17 years from 1990 and onwards contributes to examine associations with enough statistical power.

The aim of this study was to examine the association between long-term sickness absence for psychiatric disorders and the risk of mortality due to all-causes, and specific-causes (CVD, cancer and suicide) during the period 1990–2007. An additional aim was to examine results for two cohorts with psychiatric long-term sickness absence in 1990 and in 2000.

## Materials and Methods

### Ethical statement

The study population was based on register linkages. In Sweden, the use of register data always needs ethical approval by the regional ethical review boards. Additionally, the data owners make a risk appraisal related with the Law on Public Disclosure and Security. In situations of already collected data, the ethical review board can often refrain to require the researcher to contact the study subjects in order to obtain their informed consent. On the basis of these standards, this study has been approved by the Regional Ethical Review Board in Stockholm, Sweden.

### The Swedish social insurance system

In Sweden, employees can receive sickness benefits from the SSIA if the work ability is reduced by medical reasons (by ¼, ½, ¾). (4) In 1990, all sickness benefit was paid from the SSIA from the first day of sickness absence. These rules were changed in 1992/93, and in 2000 the employer financed the sick pay for the first 14 days (the employer period) and the employee did not receive any payment for the first day of sickness absence (waiting day). After the employer period, sickness benefit was paid by the SSIA, and there was no upper time limit for sickness absence during the study period. [11]–[12] From 1992 a rehabilitation benefit could be received from the SSIA during vocational rehabilitation. [4], [12] All blue collar workers and all employees within the municipalities and county councils in Sweden are provided additional sick leave insurance from AFA Insurance above the statutory insurances from the SSIA. [13]

### Study population, design and register linkages

This prospective cohort study was based on employed persons (minimum 1 hours' work/week in November each year) in the Swedish municipalities and county councils in the ages 16–60 years, who lived in Sweden on December 31, 1990 and 2000. These were 1,159 822 individuals in 1990, and 962,448 persons in 2000. The longitudinal integration database for health insurance and labour market studies (LISA) [14] from Statistics Sweden was used to identify the cohorts, and for information on socio-demographic factors, for sick leave/no sick leave (all-causes) according to the Swedish Social insurance agency (SSIA), and migration during follow-up. The information from LISA was linked to data on psychiatric long-term sickness absence from AFA Insurance (the AFA database), as well as the national inpatient register and the cause of death register from the Swedish National board of health and welfare. From 1987, the inpatient care register covers information on all individual hospital discharges in Sweden. [15] The cause of death register contains data on mortality of persons who were registered in Sweden. [16] The cause of death register and the national inpatient care register include diagnoses given by the physician.

### Exclusion criteria

We excluded employees with long-term psychiatric sickness absence according to the AFA-database at baseline but without registered sick-leave according to LISA during the same year (in 1990, n = 1), (in 2000, n = 691). Individuals not exposed to psychiatric long-term sickness absence and not part of the reference category (no registered sick leave) (1990, n = 911,872), (in 2000, n = 184,993) were excluded. Due to the employer period introduced in 1992, the reference group with no registered sick leave contains a much larger group in 2000 (including 0–14 sickness absence days) than in 1990 (0 sickness absence days). Individuals who died at baseline (1990, n = 0), (2000, n = 1); and persons with missing values on the covariates (1990, n = 1,175), (2000, n = 1,085) were also excluded. After final exclusions of individuals with disability pension in 1990 (n = 1,784) and 2000 (n = 11,218), as well as of persons with rehabilitation benefit among those in the reference group (n = 323) according to LISA in 2000, our study populations comprised 244,990 individuals in 1990 and 764,137 individuals in 2000.

### Sickness absence

The exposure variable was measured as having at least one new spell of sickness absence (full- or part-time) for psychiatric disorders exceeding 90 days from the AFA Insurance database that started at baseline (during 1990 and 2000, respectively). The reference group contains individuals (employed in November the respective years) with no registered sick leave due to any cause according to LISA in the same years. The codes on psychiatric disorders in the AFA Insurance database are based on the International Classification of Diseases (ICD-9, ICD-10) and derived from sickness certificates issued by physicians. [13] We have used an overall measure of psychiatric sickness absence coded at AFA Insurance.

### Mortality

Underlying diagnoses of mortality were coded according to ICD-9 and ICD-10. Diagnoses from ICD-9 have been converted to diagnoses according to ICD-10, using converting systems recommended by the National Board of Health and Welfare. [16] All-cause mortality and mortality due to following causes were analysed: cardiovascular diseases (ICD-9 codes 390-459, ICD-10 codes I00-I99), cancer (ICD-9 codes 140–208, ICD-10 codes C00–C97), smoking-related cancer; oral cavity (ICD-9 codes140–141 and 143–149, ICD-10 codes C00–C06 and C09–C14), esophagus (ICD-9 code 150, ICD-10 code C15), pancreas (ICD-9 code 157, ICD-10 code C25), respiratory and intrathoracic organs (ICD-9 codes 160–163, ICD-10 codes C30–C34 and C38), and urinary tract (ICD-9 codes 188–189, ICD-10 codes C64–C68) [6], and non-smoking related cancer. Suicide as a cause of death included undetermined causes of deaths in order to account for time- and regional differences in ascertainment routines. [17] The following ICD- codes on suicide and undetermined causes of deaths were combined: ICD-9 codes E950–E959 and E980–E989 and ICD-10 codes X60–X84 and Y10–Y34. A sensitivity analysis revealed comparability between the estimates for uncertain and certain suicide. This combined measure is hereafter referred to as ″suicide″.

### Covariates

Analyses were adjusted for the following socio-demographic factors at baseline (1990 and 2000): age (16–26 years [reference group], 27–37 years, 38–48 years, or 49–60 years); sex [male as the reference group]; education (high educational level: >12 years [reference group], medium educational level: 10–12 years, or low educational level: 0–9 years); country of birth (Sweden [reference group], other Nordic countries, other European countries, or rest of the world); family situation (married with/without children living at home [reference group comprises married with children], unmarried with/without children living at home, divorced/separated/widowed and with/without children living at home).

Adjustments were also made for inpatient care preceding and including baseline (1988–1990 and 1998–2000). We controlled for inpatient care due to somatic and psychiatric diagnoses in order to examine the effect of adjustment for previous inpatient care contacts on a potential association of sickness absence due to psychiatric diagnoses with mortality. The health care measures were categorized as: a) 0 hospitalization day due to the specific diagnoses [reference group], b) ≤ median hospitalization days in specific diagnoses, and c) > median hospitalization days in specific diagnoses. Hospitalization for suicide attempts was dichotomised. The median duration of inpatient care in 1988–1990 and 1998–2000, respectively, due to psychiatric diagnoses was 15 days and 8 days; due to cancer diagnoses 8 days and 5 days; due to cardiovascular diseases 4 days and 3 days; and due to all somatic diagnoses 5 days and 3 days, respectively. The analyses of all-cause mortality and suicide deaths were controlled for inpatient care due to somatic diagnoses, psychiatric diagnoses and suicide attempt. In the analyses of CVD and cancer, adjustments were made for inpatient care due to the diagnoses related to the outcome (CVD and cancer), other somatic diagnoses and psychiatric diagnoses.

### Statistical analyses

We used flexible parametric survival models [18] to test if long-term sickness absence for psychiatric disorders that started in 1990 was associated with all-cause and cause-specific mortality with a follow-up from January 1, 1991 to December 31, 2007. Additional analyses were carried out using two different cohorts of psychiatric long term sickness absence namely in 1990 with follow-up for mortality 1991–1997, as well as in 2000 with mortality 2001–2007. Individuals were censored during follow-up when emigrated or dying due to other causes than the cause-specific mortality examined. We used four internal knots (at centiles 20, 40, 60 and 80) for the baseline hazard, and two internal knots (at centiles 33, 67) for the time-dependent models. We stratified analyses by sex when the likelihood-ratio test indicated statistically significant interaction with sex. The results are presented as crude and adjusted hazard ratios (HR) with 95% confidence intervals (CI). Analyses were performed by using the statistical package STATA version 11.

## Results

### Descriptive statistics

The baseline characteristics of the employees are shown in table 1. There were more individuals with psychiatric long-term sick-listing and without registered sick leave during the more recent exposure year in 2000 than in 1990. Of the 244,990 and 764,137 individuals comprising the study base in 1990 and 2000, respectively, 2,351 individuals in 1990 and 7,719 persons in 2000 had at least one new spell of long-term psychiatric sickness absence. The greatest proportion of employees with psychiatric long-term absence and without registered sick leave in 1990 and in 2000 were women, older than 37 years of age, born in Sweden, had attained medium or high educational level, and were married with at least one child living in the household. In 1990 and 2000, the proportion of males and younger (16–26 years old) employees, respectively, was higher among those without sick leave (in 1990: 29.4%; 21.4%, in 2000: 22.3%; 11.2%) than among individuals with psychiatric long-term sickness absence (in 1990: 14.3%; 6.5%, in 2000: 10.8%, 1.9%). Additionally, in 1990 there was a larger percentage of employees with a high educational level and who were married with at least one child living at home, respectively, among those with no registered sick leave (38.1%; 39,9%) compared with psychiatric sickness absentees (29.5%; 29.8%). According to the AFA Insurance register (data not shown) 349 individuals (14.84%) with psychiatric long-term sickness absence in 1990, and 36 persons (0.01%) in the reference group with no registered sick leave during the same year, had psychiatric long-term sickness absence that started before baseline in 1989. Additionally, 495 persons (6.41%) of those individuals with psychiatric sickness absence in 2000, and 464 individuals (0.06%) in the reference group in 2000, had psychiatric long-term sickness absence starting before baseline in 1999.

**Table 1:** Descriptive statistics at baseline (1990 and 2000) among individuals with long-term sickness absence for psychiatric disorder (LTSAP) and no registered sickness absence (SA).

	1990		2000	
	No SA, N (%)	LTSAP, N (%)	No SA, N (%)	LTSAP, N (%)
All	242639 (99.0)	2351 (1.0)	756418 (99.0)	7719 (1.0)
***Sex***				
Men	71384 (29.4)	336 (14.3)	168713 (22.3)	831 (10.8)
Women	171255 (70.6)	2015 (85.7)	587705 (77.7)	6888 (89.2)
***Age group (years)***				
16–26	51894 (21.4)	153 (6.5)	84432 (11.2)	148 (1.9)
27–37	55424 (22.8)	721 (30.7)	179453 (23.7)	1613 (20.9)
38–48	78909 (32.5)	933 (39.7)	242492 (32.1)	3112 (40.3)
49–60	56412 (23.2)	544 (23.1)	250041 (33.1)	2846 (36.9)
***Country of birth***				
Sweden	221092 (91.1)	2101 (89.4)	685642 (90.6)	7108 (92.1)
Other Nordic	9476 (3.9)	134 (5.7)	24722 (3.3)	291 (3.8)
Other European	5397 (2.2)	74 (3.1)	19965 (2.7)	178 (2.3)
Rest of the world	6674 (2.8)	42 (1.8)	26089 (3.4)	142 (1.8)
***Education (years)***				
High (>12)	92480 (38.1)	693 (29.5)	354600 (46.9)	3956 (51.3)
Medium (10–12)	97851 (40.3)	1214 (51.6)	332372 (43.9)	3243 (42.0)
Low (0–9)	52308 (21.6)	444 (18.9)	69446 (9.2)	520 (6.7)
***Family situation***				
Married*	106748 (39,9)	701 (29.8)	276020 (36.5)	2816 (36.5)
Married**	31488 (13.0)	331 (14.1)	116499 (15.4)	1167 (15.1)
Unmarried*	51869 (21.4)	306 (13.0)	129258 (17.1)	1131 (14.7)
Unmarried**	34692 (14.3)	416 (17.7)	139891 (18.5)	924 (12.0)
Divorced/widowed*	8376 (3.5)	296 (12.6)	46214 (6.1)	906 (11.7)
Divorced/widowed**	9466 (3.9)	301 (12.8)	48536 (6.4)	775 (10.0)

* With children,

** Without children.

### Study period 1990–2007

Employees with a new spell of long-term sickness absence due to a psychiatric disorder in 1990, were at increased risk of mortality from 1991 to 2007 due to all- and specific-causes compared to the reference group with no registered sick leave (table 2). These estimates were reduced (except for suicide) after adjustment for age, sex, education, country of birth, and family situation (model 2). After additional adjustment for inpatient care due to somatic disorders, the estimates was increased for mortality due to all-causes (HR 2.29, 95% CI 2.0–2.6), CVD (HR 1.78, 95% CI 1.3–2.4), cancer (HR 1.22, 95% CI 1.0–1.5), and suicide (HR 12.16, 95% CI 8.9–16.7). In the final model, when adjusting also for inpatient care due to psychiatric disorders, the increased risk remained significant for all-cause mortality (HR 1.56, 95% CI 1.3–1.8), CVD (HR 1.35, 95% CI 1.0–1.9) and suicide (HR 3.84, 95% CI 2.4–6.1).

**Table 2:** Long-term sickness absence for psychiatric disorder (LTSAP) (1990) and mortality (all-cause, cardiovascular disease (CVD), cancer, suicide) (1991–2007).

		Model 1	Model 2	Model 3	Model 4*
	Deaths	HR (95% CI)	HR (95% CI)	HR (95% CI)	HR (95% CI)
***All-cause***					
No SA	9525	1	1	1	1
LTSAP	232	2.59 (2.3–2.9)	2.35 (2.1–2.7)	2.29 (2.0–2.6)	1.56 (1.3–1.8)
***CVD***					
No SA	2301	1	1	1	1
LTSAP	40	1.85 (1.3–2.5)	1.81 (1.3–2.5)	1.78 (1.3–2.4)	1.35 (1.0–1.9)
***Cancer***					
No SA	5113	1	1	1	1
LTSAP	74	1.54 (1.2–1.9)	1.35 (1.1–1.7)	1.22 (1.0–1.5)	1.07 (0.8–1.4)
***Smoking-related cancer***					
No SA	1440	1	1	1	1
LTSAP	23	1.70 (1.1–2.6)	1.47 (1.0–2.2)	1.36 (0.9–2.1)	1.03 (0.6–1.6)
***Not smoking- related cancer***					
No SA	3673	1	1	1	1
LTSAP	51	1.47 (1.1–1.9)	1.30 (1.0–1.7)	1.18 (0.9–1.5)	1.09 (0.8–1.5)
***Suicide***					
No SA	423	1	1	1	1
LTSAP	47	11.73 (8.7–15.8)	12.02 (8.8–16.5)	12.16 (8.9–16.7)	3.84 (2.4–6.1)

Flexible parametric survival models with hazard ratios (HR) and 95% confidence intervals (CI). The reference group comprises those with no registered sickness absence (SA) (1990). Model 1: Crude; Model 2: Adjusted for age, sex, education, country of origin, family situation; Model 3: Adjusted for the covariates in model 2 and inpatient care due to somatic diagnoses; Model 4: Adjusted for the covariates in model 3 and inpatient care due to psychiatric diagnoses. *All-cause mortality and suicide deaths adjusted for the covariates in model 3 and inpatient care due to psychiatric diagnoses and suicide attempt.

### Hazard ratio over time

**Figure 1:**
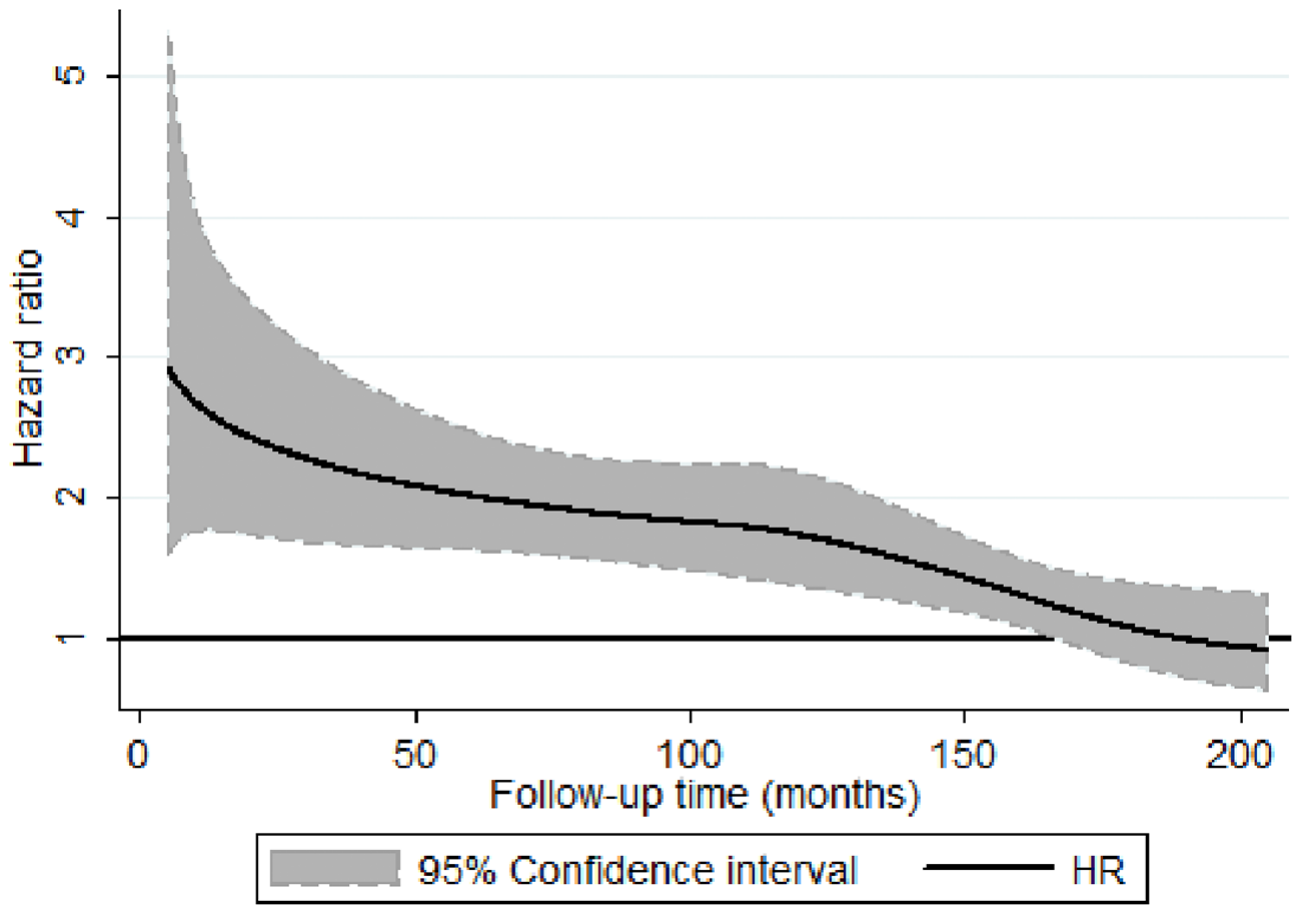
Long-term sickness absence for psychiatric disorders (1990) and all-cause mortality (199106–200712). Hazard ratio (HR) and 95% confidence interval (CI) (HR = black line and CI = shaded area) from time-dependent flexible parametric survival model adjusted for age, sex, education, country of origin, family situation, and previous inpatient care. Black line at vertical number 1 means no association.

Figure 1 shows a significantly elevated risk of all-cause mortality during most of the time period from 1991 to 2007, that decreased over time among psychiatric long-term sick-listed compared with those with no sick leave in 1990. After adjustment for all covariates, there was a 2.62 times increased risk (95% CI 1.8–3.9) after 1 year (12 months) and a 1.84 times increased risk (95% CI 1.5–2.2) after 8 years (96 months), of all-cause mortality. An elevated mortality risk, decreasing over time, could also be seen for suicide, cancer and CVD during this period, although the increased risk for cancer and CVD was not statistically significant in the fully adjusted model (data not shown).

### Study periods 1990–1997 and 2000–2007

Long-term psychiatric sickness absence in 1990 was associated with an increase in risk of mortality due to all-causes, cancer and suicide considering the follow-up period 1991–1997 (table 3, model 1, HR ranged from 1.85 to 15.59). When adjusting for all covariates the excess risk remained significant for all-cause (HR 2.00, 95% CI 1.5–2.6) and suicide mortality (HR 4.77, 95% CI 2.4–9.3).

**Table 3:** Long-term sickness absence for psychiatric disorder (LTSAP) (1990 and 2000) and mortality (all-cause, cardiovascular disease (CVD), cancer, suicide) (1991–1997 and 2000–2007).

	1990			2000		
		Model 1	Model 2*		Model 1	Model 2*
	Deaths	HR (95% CI)	HR (95% CI)	Deaths	HR (95% CI)	HR (95% CI)
*All-cause*						
No SA	2238	1	1	6795	1	1
LTSAP	75	3.49 (2.8–4.4)	2.00 (1.5–2.6)	131	1.90 (1.6–2.2)	1.47 (1.2–1.7)
*CVD*						
No SA	527	1	1	1256	1	1
LTSAP	<10	-	-	26	2.04 (1.4–3.0)	1.83 (1.2–2.7)
*Cancer*						
No SA	1182	1	1	3901	1	1
LTSAP	21	1.85 (1.2–2.8)	1.18 (0.7–1.9)	64	1.61 (1.3–2.1)	1.33 (1.0–1.7)
*Smoking-related cancer*						
No SA	311	1	1	1294	1	1
LTSAP	<10	-	-	21	1.60 (1.0–2.4)	1.44 (0.9–2.2)
*Not smoking- related cancer*						
No SA	871	1	1	2607	1	1
LTSAP	17	2.03 (1.3–3.3)	1.46 (0.9–2.4)	43	1.62 (1.2–2.2)	1.27 (0.9–1.7)
*Suicide*						
No SA	167	1	1	429	1	1
LTSAP	25	15.59 (10.2–23.7)	4.77 (2.4–9.3)	16	3.67 (2.2–6.0)	2.15 (1.3–3.7)

Flexible parametric survival models with hazard ratios (HR) and 95% confidence intervals (CI). The reference group comprises those with no registered sickness absence (SA) (1990 and 2000). Model 1: Crude; Model 2: Adjusted for age, sex, education, country of origin, family situation, and inpatient care due to somatic diagnoses and psychiatric diagnoses. *All-cause mortality and suicide deaths adjusted for age, sex, education, country of origin, family situation, and inpatient care due to somatic diagnoses, psychiatric diagnoses and suicide attempt.

Employees with long-term sickness absence due to a psychiatric disorder in 2000 were at increased risk of mortality from 2001 to 2007 due to all- and specific-causes compared to the reference group (table 2, model 1, HRs ranged from 1.60 to 3.67). After full adjustment for all covariates including inpatient care due to psychiatric disorders, the estimates was increased for all-cause mortality (HR 1.47, 95% CI 1.2–1.7), CVD (HR 1.83, 95% CI 1.2–2.7), cancer (HR 1.33, 95% CI 1.0–1.7), and suicide (HR 2.15, 95% CI 1.3–3.7).

### Hazard ratio over time

For the period 2001–2007 there was an increased risk of mortality that diminished over time among employees with long-term sickness absence for a psychiatric disorder in 2000. After adjustment for all covariates, the increased risk of overall mortality remained significant for about half of the period from 2001 to 2007. The HR for all-cause mortality was 3.72 (95% CI 2.7–5.1) after 1 year (12 months), and 1.35 (95% CI 1.0–1.8) after 3.5 years (42 months) in the fully adjusted model. An elevated risk, decreasing over time, could also be seen for CVD, cancer and suicide deaths (data not shown).

### Differences in results between women and men

We found significant interaction with gender for the association of long-term sickness absence for psychiatric disorders with all-cause mortality in the final multivariate model, as indicated by the likelihood-ratio test for the period 1990–2007 (p = 0.043), and for the period 2000–2007 (p = 0.007). Stratified analyses (not reported) showed different risk estimates for women and men during these periods. In the fully adjusted model, psychiatric absence in 1990 was associated with an increased risk for all-cause mortality during 1991–2007 among women (HR 1.47, 95% CI 1.2–1.8) and men (HR 1.92, 95% CI 1.4–2.7). Moreover, the HR for all-cause mortality in the multivariate model was 1.30 (95% CI 1.0–1.6) in women, and 2.15 (95% CI 1.6–3.0) in men considering the period 2000–2007.

## Discussion

### Main findings

Long-term sickness absence (exceeding 90 days) for psychiatric disorder was associated with an increased risk of mortality due to all-causes as well as deaths due to cardiovascular disease, cancer, and suicide. For both cohorts 1990 and 2000 estimates point in the same direction. After adjustment for a number of socio-demographic variables as well as somatic and psychiatric inpatient care, estimates remained significant for all-cause mortality and all specific causes of death in the cohort 2000, and for all-cause and suicide deaths in the cohort 1990.

### Comparison with other studies, and possible mechanisms

Long-term psychiatric sickness absence was associated with an increased risk of all-cause mortality. For the time period 1990–2007 there was a 1.6 times increased mortality risk after adjustment for socio-demographic variables and previous inpatient care due to psychiatric and somatic disorders. Moreover, we found a 1.5 times increased risk of all-cause mortality in the multivariate model during 2000–2007. These results were in line with a 1.9 and 1.7 times increased risk, respectively, of mortality due to all causes in the above mentioned studies by Head et al [5] and Mittendorfer-Rutz et al. [7] The Swedish sickness benefit system is intended to provide financial security [4] and enables rest for the patient in order to regain health and work ability. [11] However, long-term sickness absence may have negative consequences on health behaviours (food habits, physical exercise and smoking) and on social relationships which might be potentially health hazards. (*E.g*. [19]–[21]) The distinction of the mortality risk associated with the psychiatric disorder from the risk associated with long-term sickness absence cannot be made in our study. Our results showed that the risk of mortality decreased over time. This is probably partly due to the fact that the effect of exposure becomes weaker over time.

In line with previous studies [6]–[7] we found an increased risk of suicide after sickness absence for psychiatric disorders. We could now replicate these findings for long-term sickness absence in two different periods of exposure (1990 and 2000) with varying levels of psychiatric long-term sick leave in the Swedish national population. More individuals were sickness absent due to psychiatric diagnoses in the 2000 cohort compared to the 1990 cohort, but psychiatric sickness absence was associated with a lower risk of suicide in 2000 than in 1990. Explanations for these findings include that there was a general decrease in suicide rates in the Swedish population over the same period. [22] Secondly, another possible explanation for this discrepancy might be that the type of patients that accounted for the increase in psychiatric sickness absence might be a group with inherently lower suicide risk. Finally, differences in the reference groups and diagnostic patterns in the two periods, but also improvements in health care might have contributed to the lower suicide risk observed in the latter period. [23]

While the association between psychiatric disorders and suicide are well known using data from different treatment settings (*e.g*. [24]–[25]), it has to be noted that the association between long-term sickness absence due to psychiatric disorders remained significantly increased after controlling for inpatient care. This draws attention to the importance of outpatient care settings with regard to appropriate treatment and follow-up as well as thorough suicide risk assessments of patient's sickness absent due to psychiatric disorders.

We found an elevated risk of CVD mortality after long-term psychiatric sickness absence. The increased risk was in line with earlier studies on individuals sick-listed for psychiatric disorders [6]–[7], as well as with previous research from other treatment settings focusing on stress, anxiety and depression. (*E.g*., [26]–[30]) The mechanisms involved might include *e.g*. inattention by the physician to physical symptoms among patients with psychiatric disorders, delayed help-seeking for medical care among depressed people, and lifestyle factors (smoking, alcohol intake, low physical activity, obesity). [7], [27] Also biological mechanisms which may link *e.g*. depression to cardiovascular disease have been reported. [27]

Psychiatric long-term sickness absence in our study was associated with an increased risk of cancer deaths. It should be noted that the risk for cancer mortality during the more recent period 2000–2007 was increased only for a few years after the psychiatric absence, and did not differ from the reference group after three years when controlling for all covariates. The lack of significant finding related to exposure in the cohort 1990 might predominantly relate to limited statistical power.

The result related to cancer mortality is in agreement with two previous studies on patients with psychiatric sick leave. [5], [7] In the study by Mittendorfer-Rutz et al [7] an increased risk of non-smoking related cancer after psychiatric sickness absence was found. In our study, there was a tendency that psychiatric long-term absence was associated with an increased risk of non-smoking related cancer in the fully adjusted model. Research from different treatment settings has suggested that psychiatric disorders predict cancer mortality, although reports on the association with cancer incidence have yielded more mixed results. (*E.g*. [31]–[33]) The mechanisms involved might include inadequate detection of somatic disorders among patients with psychiatric disorders, delays in treatment seeking due to depression which might result in later detection of cancer, lifestyle factors, [7], [31] as well as biological mechanisms such as dysfunctional HPA-axis, and mechanisms related to the immune function which are related to stress and depression and can potentially influence cancer progression. [34] Future studies are warranted to investigate if psychiatric sickness absence can even predict cancer incidence besides cancer mortality.

Long-term psychiatric sickness absence was associated with an increased risk of mortality among both women and men. However, we found a significant interaction with gender for the association with all-cause mortality in the fully adjusted model. Stratified analyses showed a 1.5 times increased risk in women and a 1.9 times increased risk in men of all-cause mortality in the fully adjusted model for the time period 1990–2007. Considering the time period 2000–2007 the HR for all-cause mortality was 1.3 in women and 2.1 in men after adjustment for all covariates. In the study by Mittendorfer-Rutz et al [7], a higher risk of CVD mortality was seen among men but not among women during the period 2005–2008. More research is needed to elucidate gender differences in health and social outcomes of long-term sickness absence due to psychiatric disorders.

Our findings suggest that long-term sickness absence for psychiatric disorders may be used as a risk indicator for premature mortality. This stresses the importance of developing treatment strategies for return to work, and to use more broadly interventions which have shown to improve health (*e.g*. [35]) in employee's sickness absent due to a psychiatric disorder.

### Strengths and limitations

To the best of our knowledge this is the first study investigating the association of long-term sickness absence (exceeding 90 days) due to psychiatric disorders with diagnosis-specific mortality during two different exposure periods (1990 and 2000) with varying national levels of psychiatric long-term absence. The strengths of this study include the large study population and the prospective cohort design with a long follow-up time of mortality after psychiatric sickness absence. Our study included employees representing many different occupations within the municipalities and county councils. The register-based data means no bias related to self-report. Exposure data on sickness absence including diagnoses; the possibility to adjust for several potential confounders; and information on mortality with nearly complete coverage are additional advantages. [16] A major strength is also the inclusion of information on inpatient care due to different diagnoses.

Limitations include that only those employees who reported psychiatric sickness absence to the insurance company were analyzed as the exposed. There is little research on the validity of sickness absence diagnoses, but one study from Sweden has shown satisfactory validity. [36] In our study we have used an overall measure of psychiatric sickness absence at the chapter level. The validity of the distinction of a sickness episode as psychiatric rather than somatic is not known, but due to the remaining stigma related to psychiatric disorders [37] the risk of misclassification of a somatic disorder as psychiatric is likely to be small. There might be some underreporting of psychiatric disorders on sickness certificates. However, this should not change our conclusion on an association between psychiatric long-term sickness absence and increased mortality risk. The study was based on employed persons in November at baseline within the Swedish municipalities and county councils, possibly with a better health than the general population [38] which might decrease the generalizability of our findings. Our study sometimes included rather few cases of death for the psychiatric sick-leave category. In the interpretation of the estimates related to the cohort in 1990 and 2000, it should be taken into consideration that there were differences in the reference-groups in 1990 (0 absence day) and 2000 (0–14 absence days).

### Conclusions

We found increased risks of all-cause and cause-specific mortality (cardiovascular diseases, cancer and suicide deaths) among employees with long-term psychiatric sickness absence.

Long-term sickness absence for psychiatric disorders may be used as a risk indicator for premature mortality.
